# Breast cancer patient experiences through a journey map: A qualitative study

**DOI:** 10.1371/journal.pone.0257680

**Published:** 2021-09-22

**Authors:** Laura Ciria-Suarez, Paula Jiménez-Fonseca, María Palacín-Lois, Mónica Antoñanzas-Basa, Ana Fernández-Montes, Aranzazu Manzano-Fernández, Beatriz Castelo, Elena Asensio-Martínez, Susana Hernando-Polo, Caterina Calderon

**Affiliations:** 1 Clinical Psychology and Psychobiology Department, Faculty of Psychology, University of Barcelona, Barcelona, Spain; 2 Medical Oncology Department Hospital Universitario Central of Asturias, Oviedo, Spain; 3 Social Psychology and Quantitative Psychology Department, Faculty of Psychology, University of Barcelona, Barcelona, Spain; 4 Medical Oncology Department, Hospital Universitario Clínico San Carlos, Madrid, Spain; 5 Medical Oncology Department, Complexo Hospitalario Universitario de Ourense, Ourense, Spain; 6 Medical Oncology Department, Hospital Universitario La Paz, Madrid, Spain; 7 Medical Oncology Department, Hospital General Universitario de Elche, Elche, Spain; 8 Medical Oncology Department, Hospital Universitario Fundación Alcorcón, Madrid, Spain; Kaiser Permanente Washington, UNITED STATES

## Abstract

**Background:**

Breast cancer is one of the most prevalent diseases in women. Prevention and treatments have lowered mortality; nevertheless, the impact of the diagnosis and treatment continue to impact all aspects of patients’ lives (physical, emotional, cognitive, social, and spiritual).

**Objective:**

This study seeks to explore the experiences of the different stages women with breast cancer go through by means of a patient journey.

**Methods:**

This is a qualitative study in which 21 women with breast cancer or survivors were interviewed. Participants were recruited at 9 large hospitals in Spain and intentional sampling methods were applied. Data were collected using a semi-structured interview that was elaborated with the help of medical oncologists, nurses, and psycho-oncologists. Data were processed by adopting a thematic analysis approach.

**Results:**

The diagnosis and treatment of breast cancer entails a radical change in patients’ day-to-day that linger in the mid-term. Seven stages have been defined that correspond to the different medical processes: diagnosis/unmasking stage, surgery/cleaning out, chemotherapy/loss of identity, radiotherapy/transition to normality, follow-up care/the “new” day-to-day, relapse/starting over, and metastatic/time-limited chronic breast cancer. The most relevant aspects of each are highlighted, as are the various cross-sectional aspects that manifest throughout the entire patient journey.

**Conclusions:**

Comprehending patients’ experiences in depth facilitates the detection of situations of risk and helps to identify key moments when more precise information should be offered. Similarly, preparing the women for the process they must confront and for the sequelae of medical treatments would contribute to decreasing their uncertainty and concern, and to improving their quality-of-life.

## Introduction

Breast cancer is the most common cancer and the one that associates the highest mortality rates among Spanish women, with 32,953 new cases estimated to be diagnosed in Spain in 2020 [1]. Thanks to early diagnosis and therapeutic advances, survival has increased in recent years [2]. The 5-year survival rate is currently around 85% [3, 4].

Though high, this survival rate is achieved at the expense of multiple treatment modalities, such as surgery, chemotherapy, radiotherapy, and hormone therapy, the side effects and sequelae of which can interfere with quality-of-life [5]. Added to this is the uncertainty surrounding prognosis; likewise, life or existential crises are not uncommon, requiring great effort to adjust and adapt [6]. This will not only affect the patient psychologically, but will also impact their ability to tolerate treatment and their socio-affective relations [7].

Several medical tests are performed (ultrasound, mammography, biopsy, CT, etc.) to determine tumor characteristics and extension, and establish prognosis [8]. Once diagnosed, numerous treatment options exist. Surgery is the treatment of choice for non-advanced breast cancer; chemotherapy, radiotherapy, and hormone therapy are adjuvant treatments with consolidated benefit in diminishing the risk of relapse and improving long-term survival [9]. Breast cancer treatments prompt changes in a person’s physical appearance, sexuality, and fertility that interfere with their identity, attractiveness, self-esteem, social relationships, and sexual functioning [10]. Patients also report more fatigue and sleep disturbances [11]. Treatment side effects, together with prognostic uncertainty cause the woman to suffer negative experiences, such as stress in significant relationships, and emotions, like anxiety, sadness, guilt, and/or fear of death with negative consequences on breast cancer patients’ quality-of-life [10, 12]. Once treatment is completed, patients need time to recover their activity, as they report decreased bodily and mental function [13], fear of relapse [14], and changes in employment status [15]. After a time, there is a risk of recurrence influenced by prognostic factors, such as nodal involvement, size, histological grade, hormone receptor status, and treatment of the primary tumor [16]. Thirty percent (30%) of patients with early breast cancer eventually go on to develop metastases [17]. There is currently no curative treatment for patients with metastatic breast cancer; consequently, the main objectives are to prolong survival, enhance or maintain quality-of-life, and control symptoms [17, 18]. In metastatic stages, women and their families are not only living with uncertainty about the future, the threat of death, and burden of treatment, but also dealing with the existential, social, emotional, and psychological difficulties their situation entails [18, 19].

Supporting and accompanying breast cancer patients throughout this process requires a deep understanding of their experiences. To describe the patient’s experiences, including thoughts, emotions, feelings, worries, and concerns, the phrase “patient voice” has been used, which is becoming increasingly common in healthcare [20]. Insight into this “voice” allows us to delve deeper into the physical, emotional, cognitive, social, and spiritual effects of the patient’s life. This narrative can be portrayed as a “cancer journey", an experiential map of patients’ passage through the different stages of the disease [21] that captures the path from prevention to early diagnosis, acute care, remission, rehabilitation, possible recurrence, and terminal stages when the disease is incurable and progresses [22]. The term ‘patient journey’ has been used extensively in the literature [23–25] and is often synonymous with ‘patient pathway’ [26]. Richter et al. [26] state that there is no common definition, albeit in some instances the ‘patient journey’ comprises the core concept of the care pathway with greater focus on the individual and their perspective (needs and preferences) and including mechanisms of engagement and empowerment.

While the patient’s role in the course of the disease and in medical decision making is gaining interest, little research has focused on patient experiences [27, 28]. Patient-centered care is an essential component of quality care that seeks to improve responsiveness to patients’ needs, values, and predilections and to enhance psychosocial outcomes, such as anxiety, depression, unmet support needs, and quality of life [29]. Qualitative studies are becoming more and more germane to grasp specific aspects of breast cancer, such as communication [27, 30], body image and sexuality [31, 32], motherhood [33], social support [34], survivors’ reintegration into daily life [13, 15], or care for women with incurable, progressive cancer [17]. Nevertheless, few published studies address the experience of women with breast cancer from diagnosis to follow-up. These include a clinical pathway approach in the United Kingdom in the early 21st century [35], a breast cancer patient journey in Singapore [25], a netnography of breast cancer patients in a French specialized forum [28], a meta-synthesis of Australian women living with breast cancer [36], and a systematic review blending qualitative studies of the narratives of breast cancer patients from 30 countries [37]. Sanson-Fisher et al. [29] concluded that previously published studies had examined limited segments of patients’ experiences of cancer care and emphasized the importance of focusing more on their experiences across multiple components and throughout the continuum of care. Therefore, the aim of this study is to depict the experiences of Spanish breast cancer patients in their journey through all stages of the disease. To the best of our knowledge, there are no studies that examine the experience of women with breast cancer in Spain from diagnosis through treatment to follow-up of survivors and those who suffer a relapse or incurable disease presented as a journey map.

A map of the breast cancer patient’s journey will enable healthcare professionals to learn first-hand about their patients’ personal experiences and needs at each stage of the disease, improve communication and doctor-patient rapport, thereby creating a better, more person-centered environment. Importantly, understanding the transitional phases and having a holistic perspective will allow for a more holistic view of the person. Furthermore, information about the journey can aid in shifting the focus of health care toward those activities most valued by the patient [38]. This is a valuable and efficient contribution to the relationship between the system, medical team, and patients, as well as to providing resources dedicated to the patient’s needs at any given time, thus improving their quality of life and involving them in all decisions.

## Methods

### Study design and data collection

We conducted a qualitative study to explore the pathway of standard care for women with breast cancer and to develop a schematic map of their journey based on their experiences. A detailed description of the methodology is reported in the published protocol “Ascertaining breast cancer patient experiences through a journey map: A qualitative study protocol” [39].

An interview guide was created based on breast cancer literature and adapted with the collaboration of two medical oncologists, three nurses (an oncology nurse from the day hospital, a case manager nurse who liaises with the different services and is the ‘named’ point of contact for breast cancer patients for their journey throughout their treatment, and a nurse in charge of explaining postoperative care and treatment), and two psycho-oncologists. The interview covered four main areas. First, sociodemographic and medical information. Second, daily activities, family, and support network. Third, participants were asked about their overall perception of breast cancer and their coping mechanisms. Finally, physical, emotional, cognitive, spiritual, and medical aspects related to diagnosis, treatment, and side effects were probed. Additionally, patients were encouraged to express their thoughts should they want to expand on the subject.

The study was carried out at nine large hospitals located in six geographical areas of Spain. To evaluate the interview process, a pilot test was performed. Interviews were conducted using the interview guide by the principal investigator who had previous experience in qualitative research. Due to the Covid-19 pandemic, all interviews were completed online and video recorded with the consent of the study participants for subsequent transcription. Relevant notes were taken during the interview to document key issues and observations.

### Participant selection and recruitment

Inclusion criteria were being female, over 18 years of age, having a diagnosis of histologically-confirmed adenocarcinoma of the breast, and good mental status. To ascertain the reality of women with breast cancer, most of the patients recruited (80%) had been diagnosed in the past 5 years. Patients (20%) were added who had been diagnosed more than 5 years earlier, with the aim of improving the perspective and ascertaining their experience after 5 years.

Medical oncologists and nurses working at the centers helped identify patients who met the inclusion criteria. Participants went to the sites for follow-up between December 2019 and January 2021. Eligible women were informed of the study and invited to participate during an in-person visit by these healthcare professionals. Those who showed interest gave permission to share their contact information (e-mail or telephone number) with the principal investigator, who was the person who conducted all interviews. The principal investigator contacted these women, giving them a more detailed explanation of the study and clarifying any doubts they may have. If the woman agreed to participate, an appointment was made for a videoconference.

A total of 21 women agreed to participate voluntarily in this research. With the objective of accessing several experiences and bolstering the transferability of the findings, selection was controlled with respect to subjects’ stage of cancer, guaranteeing that there would be a proportional number of women with cancer in all stages, as well as with relapses.

### Data analysis

The data underwent qualitative content analysis. To assure trustworthiness, analyses were based on the system put forth by Graneheim, and Lundman [40]. Interviews were transcribed and divided into different content areas; units of meaning were obtained and introduced into each content area; meaning codes were extracted and added; codes were categorized in terms of differences and similarities, and themes were created to link underlying meanings in the categories. All members of the research team (core team, two medical oncologists, three nurses and two psycho-oncologists) reviewed the data and triangulated the outcomes between two sources of data: qualitative data from the interview and non-modifiable information, such as sociodemographic (i.e., age, marital status, having children) and clinical (i.e., cancer stage and surgery type) data. Following this process, we reached saturation of the interview data by the time we had completed 21 interviews.

### Ethical considerations

This study was performed in accordance with the ethical standards of the Declaration of Helsinki, and its subsequent amendments. The study was approved by the Research Ethics Committee of University of Barcelona (Institutional Review Board: IRB00003099) and supported by the Bioethics Group of the Spanish Society of Medical Oncology (SEOM) 2018 grant. All participants received a written informed consent form that they signed prior to commencing with the interviews and after receiving information about the study.

## Results

### Patient baseline characteristics

In total, 21 women with a mean age of 47 years (range, 34 to 61) were interviewed. Most of the study population was married (66.7%), had a college education (66.7%), and had 2 or more children (42.9%). All cancer stages were represented, up to 23.8% tumor recurrence, and most of the primary cancers had been resected (95.2%) (see Table 1).

**Table 1:** Socio-demographic and clinical characteristics of study participants (N = 21).

Variables	N (%)
**Age**	
≤45	10 (47.6%)
>46	11 (52.4%)
**Educational level**	
< High school	3 (14.3%)
High school graduate	4 (19.0%)
College graduate	14 (66.7%)
**Marital Status**	
Single	4 (19.0%)
Married	14 (66.7%)
Divorced	3 (14.3%)
**Children**	
0	4 (19.0%)
1	8 (38.1%)
≥2	9 (42.9%)
**Employment Status**	
Employed	8 (38.1%)
Unemployed/On leave	2 (9.5%)
Disability (or pending)	8 (38.1%)
Early retirement	3 (14.3%)
**Cancer stage**	
I	4 (19.0%)
II	4 (19.0%)
III	5 (23.8%)
IV	3 (14.3%)
Relapse	5 (23.8%)
**Surgery Type**	
Tumorectomy	8 (38.1%)
Mastectomy	12 (57.1%)
Unresected	1 (4.8%)

### Description of the breast cancer patient journey

The women diagnosed with breast cancer describe the journey as a process tremendously affected by the different medical stages. Each stage has its own characteristics that condition the experiences, unleashing specific physical, emotional, cognitive, and social processes. Additionally, the patients perceive this entire process as pre-established journey they must undertake to save their life, with its protocols based on the type and stage of cancer.

“*People said to me*, *‘What do you think*?*’ and I answered that there was nothing for me to think about because everything is done*, *I have to go on the journey and follow it and wait to see how it goes” (Patient 6)*

**Fig 1:**
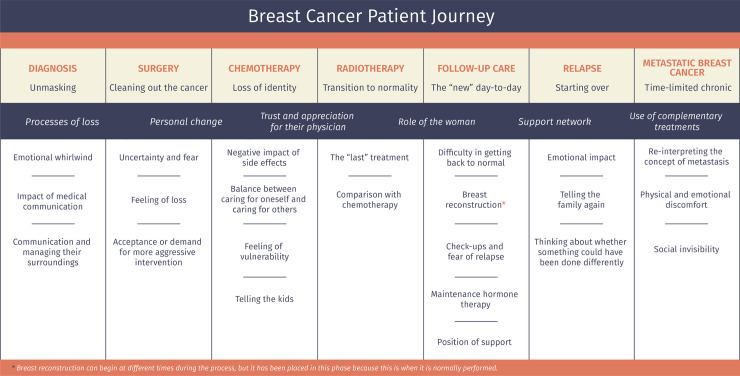


Fig 1 displays the various phases of the journey that patients with breast cancer go through; nevertheless, each woman will go through some or others, depending on their type of cancer.

#### Throughout the entire patient journey

*Processes of loss and reinterpretation of the new circumstance*. What stands out the most in the process these women go through during the diagnosis and treatment of breast cancer is loss; specifically, the loss of health and a reinterpretation of the new circumstance and the new bodily reality. In the most extreme cases, the loss of health emerges with the fear of death that many women report at the time of diagnosis or during treatment, due to the distress generated. The loss of identity seems to be related to the evolutionary (existential) moment in which the woman is; there are patients who report feelings of disability or loss of attractiveness, or fear of not being able to get pregnant in the future, especially the youngest.

*I felt a terrifying fear and thought*, *“You have cancer you tell yourself*, *you’re going to die tomorrow*.” *(Patient 6)*

*I feel like after the hysterectomy*, *as a woman*, *I no longer have anything*, *only the physical*. *Sure*, *I look great*, *but I tell myself that it’s just a shell*, *the shell I inhabit*, *because as a woman*, *I only have one breast left*. *(Patient 6)*

*At that moment*, *I had to make the decision that I was no longer going to be a mother*. *(Patient 14)*

*Personal change*. Most of the women report that with the diagnosis of breast cancer, their life stands still and from that point forward, a different journey begins. The sole focus on this journey is the disease and its implications. During all those months, the patients stop working; they focus on their medical treatments, and reflect a lot on their current situation and on life. Most of the participants state, especially those who have already been discharged, that they know themselves better now; they take better care of themselves, and they enjoy their day-to-day and the small moments more, making the most of their time, with more initiatives and fewer trivial complaints.

*Clearly*, *you’re not the same person you were before; I don’t think she’ll ever come back; your mindset changes completely and I have sequelae from all the treatments*. *(Patient 1)*

*I re-think wasting energy on lost causes; what’s more*, *I’ve also learnt to say no*. *If I’m not in the mood to go somewhere*, *I just say no*. *(Patient 7)*

*I take much more advantage of the present now*, *because you realize that things can change on any given day*. *(Patient 3)*

*Trust and appreciation for their physician*. Most of the interviewees stated that they fully trusted the doctors who care for them, without question or objection to the treatments proposed. They reported that, as they go forward, they discuss the tests and treatments that are going to be performed, as well as possible side effects. Several stated that they are unaware of the stage of their cancer; similarly, most also do not know the benefits expressed in X% of the treatments. A few of the participants claimed that they did talk in detail about the different types of treatments with their oncologists, that they had sought another opinion, and one of them even reported having decided to stop chemotherapy, which was very hard for her, given her physician’s insistence that she continue.

*The truth is that the oncologist didn’t say much about percentages; what she told me were the steps that I had to take; I thoroughly trusted her and she gave me a lot of peace of mind*. *(Patient 5)*

*I told him*, *“I’m going to do whatever you tell me to*.*” It never occurred to me to dispute whatever the oncologist might tell me*. *I was willing to do whatever was needed*. *(Patient 8)*

Most of the women, at some point during the interview, state that they are grateful for the care they received and that, within the seriousness of their situation, there is a treatment for their condition.

*I am super grateful for the treatment I’ve received and with the doctors assigned to me*. *(Patient 2)*

*I’m very lucky; I’m only on my second line of treatment for metastasis and I’ve got a lot more ahead of me*, *but I consider myself lucky and I believe things are going very well*. *(Patient 20)*

*Role of the woman*. We can see that the women adopt a role of care-givers and managers of their surroundings. They worry about the disease negatively affecting the people around them, which is why they make an effort to manage the family’s activity for when they can’t do it and they try to avoid being a physical burden or cause emotional distress to the people around them.

*I was very strong*; *I made everything easy for people*, *but making it very easy*, *doesn’t mean that it was easy for me*, *but that I made it easy for everyone*. *(Patient 8)*

*I didn’t want to worry anyone because that’s just the way I am*, *I push forward and that’s that*. *(Patient 5)*

*Support network*. In all cases, the family appears to be one of the elements that is most involved in the disease process. Within the family, the partner deserves special mention. The testimonies in this regard reveal a wide spectrum of possibilities that range from the feeling of having had great support to a lack of attention and understanding that, in many situations, causes the relationship to be strained or to end. Friends tend to appear more occasionally.

*I can’t complain about my husband; he was up to the challenge*, *very attentive toward me and he fully understood how I was feeling*; *I felt very supported*. *(Patient 14)*

*We’ve had a period of a lot of arguing; I’ve had to sit down with him and tell him that life had changed for me*. *(Patient 18)*

*I had a partner I had lived with for five and a half years and he told me*, *literally*, *that he looked at me like a little sister*, *no longer as a woman*, *and he left me*, *and that hurt me tremendously*. *(Patient 6)*

On the other hand, many patients commented on the importance of social media, where they have met people in the same situation as them. They report feeling understood and in good company; likewise, they commented on the importance of being able to share their doubts and get to know about other experiences.

*It’s a situation that only someone who has gone through can understand; you can have all the good intentions in the world*, *but if you haven’t gone through it*, *you can’t even begin to understand*. *(Patient 8)*

*Use of complementary treatments*. Most patients follow conventional medical treatment. However, many resort to other disciplines that help them improve their quality-of-life, like dietary changes, getting more exercise than usual, visits to a psychologist or physical therapist, or using other integrative therapies, such as acupuncture, yoga, reiki, flowers of Bach, homeopathy, cannabis, or meditation.

*I started to read a whole bunch of books to see what I could do to take care of myself in terms of nutrition and exercise*; *you consider everything you can do*. *(Patient 5)*

#### Diagnosis/unmasking

This phase encompasses the time from when the woman detects some symptom or goes to a check-up until the medical diagnosis is made. For the woman, this is a time of a series of tests and results. We have observed that the procedures, especially the healthcare professionals that deal with the patients, and the timing vary, depending on the medical center where they are being cared for. Emotionally, this is one of the most complicated stages.

*Emotional whirlwind*. The wait to obtain test results has a huge emotional impact for the women, given that it is a time of great uncertainty and fear.

*An entire month with all the anguish of finding out if you have something*. *(Patient 3)*

*The worst part is waiting 15 days to find out the magnitude of the tragedy*, *if it’s throughout your entire body or only in your breast; you go through a brutal emotional whirlwind; the wait is horrible because there’s nothing else you can do*, *so that anguish that you carry inside is dreadful; it was hell for me*. *(Patient 10)*

Additionally, the interviewees described many other emotions that included fear of death, fear of having no time, feeling of unreality, rage, anger, sadness, avoidance, denial…

*The first thing I thought was that I was going to die and that I wouldn’t finish watching my children [grow up]; my father had died of lung cancer 25 years ago*. *(Patient 9)*

*My only aim was to get back to normal*, *as if there were nothing wrong*. *(Patient 4)*

*You have a lot of conflicting feelings; you wish this weren’t happening; you want to run away*, *but you say*, *“Where am I going to run to*?*”*. *(Patient 14)*

*Impact of medical communication*. Several women comment that, when given the diagnosis, they dissociate because of the emotional impact and that they don’t listen to all the information that the medical professional is giving them.

*I remember that she talked and talked*, *but I didn’t know what she was saying until she said*, *“Isabel*, *you’re going to be cured*, *okay*?”. *(Patient 9)*

During the diagnostic testing, the women are highly sensitive to the healthcare professionals’ words and gestures.

*I looked at the face of the person who was doing the mammogram and that’s when I started to imagine the worst*. *(Patient 20)*

*I say to them*, “*But*, *is there a solution to this*?*”*, *and they say to me*, *“Don’t worry*, *I’m sure there is a solution*.*” That “sure” is etched in my mind*. *(Patient 10)*

*Communication and managing their surroundings*. After the diagnosis, the patients feel that they have to tell the people around them about their situation, especially those closest to them, the family. They all agree on how hard it is to share. Normally, the people it’s hardest to tell are their mother and their children. When they do, they try to put the most positive spin on it possible, in an attempt to keep them from worrying.

*You no longer think only about yourself*, *you think*, *“Good grief*, *now I’ve got to tell my mother*.” *It’s hard*. *(Patient 16)*

*I wanted to tell my kids the way I say things*, *always trying to look for the upside*, *and positive*, *although it was hard*, *but*, *anyway*, *in the end*, *it went well*. *When I finished*, *my husband told me*, *“You’ve convinced me that it’s no big deal*.” *(Patient 9)*

*I told my son*, *“Son*, *don’t cry*, *your mom’s going to get over this*, *this is nothing*.” *(Patient 1)*

During this period, the women contemplate how their situation will affect their surroundings and they try to organize it as much as possible.

*I devoted myself to planning everything*, *to organizing what to do with my daughter*, *and to thinking about work*, *too*, *how I had left things at work*. *(Patient 4)*

#### Surgery/cleaning out the cancer

*Uncertainty and fear*. The participants express that before going into surgery, they are told about the kind of procedure that will be done, but that, depending on what they find and the analysis, it may change. In light of this, they exhibit an enormous feeling of uncertainty and fear. In addition, many voice concern about how the surgery will go.

*They tell you conservative surgery*, *but if we open up and see something we didn’t see on the tests*, *then everything could change*. *(Patient 10)*

*Aside from the anesthesia*, *that I’m terrified of*, *you spend several hours in surgery and you don’t really know how things will go; when they clean it out*, *they analyze it*, *and you go into the operating room and you don’t know what can happen*. *(Patient 9)*

*Feeling of loss*. Considering that the breast is associated with an intimate, feminine part [of their body], many women experience the operation as a loss. This loss is more acute if the operation is a mastectomy and there is no reconstruction at the same time. The loss also involves a loss of identity, compounded by the side effects of chemotherapy, such as hair loss. The interviewees who had undergone mastectomy say that following surgery, when the bandaging is removed and the scar is revealed, is one of the most critical moments, which is why they express difficulty in managing it and appreciate the caring assistance from the professionals.

*It is identification with yourself*, *you know*, *it’s what you’ve seen in the mirror*, *what you think you’re like and*, *suddenly*, *you’re no longer like that; there’s an incredible personal crisis because you no longer recognize what you’re seeing*. *(Patient 11)*

*I closed my eyes and I removed the bandaging and I didn’t dare look* … *with my eyes*, *I imagined the worst*. *(Patient 12)*

*Acceptance or demand for more aggressive intervention*. The patients perceive the surgery as essential to recovering their health, which is why the process is widely accepted. Some patients who demand a more invasive intervention, normally a bilateral mastectomy, do so because that way, they feel safer with respect to a possible relapse, as well as more comfortable esthetically.

*If they have to remove my breast*, *let them take it; what I want is to get better*. *(Patient 16)*

*They say that I am in full remission*, *so they only removed the lump*, *but at first*, *I said that I wanted my whole [breast] removed*; *then they assessed how to do it*. *(Patient 13)*

*They told me that I had a genetic mutation and more possibilities of developing breast cancer and*, *since I felt such rejection toward my remaining breast*, *I decided to get rid of that one*, *too*. *(Patient 20)*

#### Chemotherapy/loss of identity

The chemotherapy phase is one of the phases that affects the women’s lives the most, because of its physical impact and how long it lasts. No differences have been found in how they experience chemotherapy depending on whether it was neoadjuvant or adjuvant.

*Negative impact of side effects*. Chemotherapy is associated with many side effects that vary from one woman to another. Many indicate that they have suffered physical discomfort, such as fatigue, dysgeusia, pain, nausea and vomiting, mucositis, diarrhea, etc.

*One day when I didn’t want to go to bed*, *I went to bed crying because I had the feeling that I wasn’t going to wake up*. *That day it was because I felt awful*. *(Patient 1)*

Furthermore, all of the women suffer hair loss, which is one of the most-feared effects. Likewise, their body hair also falls out, especially on their face, and their weight fluctuates. All of these changes lead to a loss of identity that is experienced as taking away from their femininity. It must be remembered that oftentimes, chemotherapy is administered after surgery, further exacerbating this physical change. On top of all that, several women comment having to decide at the beginning of treatment whether to freeze their eggs or not; at that moment, many of them forfeit the possibility of becoming a mother or of becoming a mother again, which also adds to this loss of femininity.

*Losing my hair was hard*, *but when it grew out again*, *I had an identity crisis*. *I didn’t recognize myself; people said I was really pretty like that*, *with my hair so short*. *I looked at myself in the mirror and I said that I’m not that woman*, *I can see that that woman is pretty*, *but it’s just that I don’t recognize myself*. *That’s not me or*, *it was like*, *I looked at myself and I didn’t recognize myself*. *That’s when I suffered a serious identity crisis*, *psychologically serious*, *but also serious because I sobbed because I looked at myself*, *but it wasn’t me*. *(Patient 6)*

*Where’s that sexy lady*, *where is she*?, *because you don’t feel good*. *I didn’t like myself at all*. *I was several sizes larger and I looked at myself and said*, *“What a monster*.*” I didn’t feel good about myself*. *(Patient 1)*

Many patients say that chemotherapy decreases their libido and dries up their mucous membranes, which is why they prefer not to have sex. For those who live as a couple, this situation can strain the relationship.

*Sexually*, *I just didn’t feel like it*, *I wasn’t in the mood; not only did I not feel like it*, *my mucous membranes were dry and*, *what’s smore*, *I just couldn’t*, *I couldn’t*, *I felt bad for my husband*, *but he said*, *“Don’t worry*.” *(Patient 16)*

Finally, some interviewees expressed a feeling of being poisoned by the treatment. These women tend to be highly focused on taking care of their body and have a very natural attitude toward life.

*I had to really work my awareness that I was poisoning myself; at night I was at home and I thought that all that red liquid was circulating through my veins … I think I even had nightmares*. *(Patient 4)*

*Balance between caring for oneself and caring for others*. The patients feel that it is time to take care of themselves, so they prioritize resting when they need it. Moreover, they worry about getting a haircut and, most of the times, they look for turbans and wigs. Some also learn how to put on make-up, which they rate as being very positive. On the other hand, those who have children or another person in their care, try to take care of them as much as they are able.

*Around 11*:*00*, *I no longer felt good*, *so I’d go to the armchair to rest and it’s like I had an angel*, *because I’d wake up a minute before I had to set the table and get lunch for my son who would be coming home from school*. *(Patient 1)*

*While I was getting chemo*, *I went with the gadget and I told myself*, *“I’m going to teach you to apply make-up; for instance*, *your eyelashes are going to fall out*. *Make a line like this*” *and at that moment when you look in the mirror*, *and we look like Fester in the Addams family*. *(Patient 13)*

*Vulnerability*. The women experience great uncertainty and feelings of vulnerability the first times they receive chemotherapy, since they don’t know what side effects they will suffer.

*With chemo*, *I started with a lot of fear and*, *later on*, *I became familiar with it little by little until the time comes when you go to the hospital like someone who’s going to pick up a bit of paper*. *(Patient 9)*

In addition, those participants who join a social network or who are more closely tied to the hospital setting, know about the relapses and deaths of people around them diagnosed with breast cancer, which makes them feel highly vulnerable.

*There are some people who leave the group because*… *it’s not like there are a lot of relapses and*, *geez*, *I think that it messes with your head*. *(Patient 13)*

*We were almost always the same people at chemotherapy*; *there was one guy who was really yellow who looked terrible and*, *there was one time when we stopped seeing him and another lady asked and the nurse said that he had died*. *(Patient 15)*

At the same time, given the physical changes, especially those that have to do with body hair, many women feel observed when they leave home.

*If I have to go out and take off my scarf because I’m hot or go straight out without any scarf on my head and whoever wants to look… let them*; *I think that it’s up to us*, *the patients*, *to normalize the situation; unfortunately*, *there are more and more cases*. *(Patient 9)*

*Telling the kids*. Since when the chemotherapy stage is going to entail many physical changes, the women look for ways to talk to their children about the treatment. Most of them comment that it is a complicated situation and all of them try to talk to their children in such a way as to protect them as much as possible.

*I asked the nurse for help before I started chemotherapy to see if she had any pointers about how to talk about this with the kids and she recommended a story*, *but when I saw it*, *I didn’t like it* … *so*, *in the end*, *I decided to do it off the cuff*. *(Patient 10)*

#### Radiotherapy/transition to normality

*The “last” treatment*. When the patient reaches radiotherapy, normally, they have already spent several months undergoing physically aggressive medical procedures, which is why they feel exhausted. There is a physical exhaustion resulting from the previous treatments and made worse by the radiation therapy. Furthermore, many women also report feeling emotionally drained by the entire process. However, this is generally accompanied by joy and relief because they feel that they are in the final stage of treatment.

*Emotionally*, *it’s a marathon that has to end up at some point*. *(Patient 10)*

*For me*, *radiotherapy was like a lull in the battle*, *with a winning mind-set*. *(Patient 4)*

*Comparison with chemotherapy*. There is a widespread perception that radiotherapy has fewer side effects than chemotherapy, although later, when they receive it, several patients suffer discomfort, above all fatigue and dizziness. Several report that at this point, they are mentally worn out and just want to be done with the process, which is why they have less information than about chemotherapy.

*I feel like radiotherapy is unknown*, *that you think it’s more “light*” *and it turns out not to be so light*. *(Patient 13)*

#### Follow-up care/the “new” day-to-day

*Difficulty in getting back to normal*. Once the patients are discharged, many feel that they need some time to recover, that it will be slow, in order to restore a more normalized pace of life. They are still working on their emotional and personal process.

*When they tell you that you have cancer*, *they make it very clear*: *you have a goal; you have some months of chemo*, *some months of radio*, *and when you finish*, *you say*, *“And now*, *what do I do*?”. *I say that because now I have to get back to my normal life*, *but I don’t feel normal*. *I still don’t feel cured*, *I’m not 100%*. *And you’re glad you’ve that you’ve finished it all and you’re alive*, *but at the same time*, *you say*, *“Gosh… this is very odd*.*” It was a very strange feeling*. *(Patient 8)*

Most patients report that their quality-of-life has diminished, due to the sequelae from the treatments. Lymphedema is one of the sequelae they name most often, although they also mention other symptoms, like digestive upset, weight issues, eye problems, scar pain, etc. The women who are on hormone therapy also suffer side effects, such as joint and muscle pain.

*I have lymphedema and*, *although I have good mobility*, *I’m a little bit weak; when I go out for dinner*, *I generally order fish*, *because I can’t always cut meat well*. *(Patient 6)*

Several interviewees also express difficulty in their affective-sexual relations. Many of them feel insecure because of all the physical changes; others have sequelae that hinder their relations, and still others are suffering symptoms of early menopause. This can cause problems in the couple and for those who don’t have a partner, suffer many complications when it comes to meeting other people.

*I haven’t had sex with my husband for 2 years because*, *it’s also really complicated to get over; I’ve gone for pelvic physical therapy; I’ve used gels*, *but nothing works*. *(Patient 8)*

*It’s taken me many months for me to have a relationship again; it’s been really hard because*, *even though everyone told me that I looked fine*, *I didn’t feel fine*. *My breast cancer had taken away all my attributes as a woman*. *(Patient 6)*

Some women also experience difficulties when it comes to returning to work. Several state that they had been fired when they went back. They also report that when interviewing for a job, it’s complicated for them because they have to explain what happened and they mention the schedule of doctor’s visits that they have. Other women comment that they’ve been given early retirement or disability.

*You go to the interview and if you tell them that you’ve had the disease*, *they look at you like you’re a weirdo*. *(Patient 13)*

*Breast reconstruction*. How reconstruction is experienced, as well as its timing, are highly contingent upon they type of reconstruction. Each one has its pros and cons, but the opinions collected with respect to the type of reconstruction have been positive.

*Although it took 18 months for the entire process to be over*, *I’m delighted with reconstruction with the expander*. *(Patient 16)*

Some patients state that after the whole process, which has been long and complicated, they prefer not to undergo reconstruction immediately. In these cases, they report having felt a subtle pressure from the outside to undergo reconstruction.

*Every time I went for my check-ups*, *they said*, *“You’re the only one left [who hasn’t undergone reconstruction]” and in the end*, *the truth is that I’m really happy because I think I look pretty*. *(Patient 12)*

*Check-ups and fear of relapse*. Check-ups are one of the times that generate most worry and insecurity. The women remark that, starting a few days before and until they receive the results of the follow-up studies, they are more anxious about the possibility of relapse.

At every check-up my legs start shaking again and my stomach is in knots, although at my last one, everything turned out okay and I’m thrilled. (Patient 6)

*During the first stage*, *I did everything I had to do and I got over it*, *but it’s a lottery*. *You can do whatever you want*, *but it’s the luck of the draw and when you start going for check-ups*, *it’s like going to play Russian roulette*. *(Patient 8)*

*Maintenance hormone therapy*. Hormone therapy is understood differently depending on age and on the major decision of whether or not to be a mother or to have another child. If the woman does not want to have more children, the treatment is accepted better. The patients who take it also report effects derived from menopause, for instance, joint pain or dry mucous membranes.

*I did notice joint pain*, *but since I exercised*, *[I felt it] much less than my fellow women*, *although*, *for instance*, *when it comes to getting up from a chair*, *you get up like an old lady*. *(Patient 10)*

*Position of support*. Several patients mention that, after discharge, they stay active on social media, they volunteer when they find out about someone or to participate in activities related to breast cancer, with the aim of being able to help other people who are in this situation.

*It’s really good to meet other people who are going through the same thing*, *so*, *now that I’ve finished*, *I like it and I always help whenever I can*, *because I can share what was good for me*. *(Patient 13)*

#### Relapse/starting over

*Emotional impact*. The diagnosis of a relapse is experienced much the same as the initial diagnosis. All of the women report fear, although they also state that they are more familiar with the processes. Other emotions emerge, such as why me, blame, disbelief, etc.

*Since they had told me that it wasn’t going to happen again*, *I believed it*, *of course*, *I wanted to believe it and it totally surprised me; I couldn’t stop crying and crying*. *(Patient 17)*

*Telling the family again*. Patients repeat that telling the family about it again, especially the children and parents, is tough and they try to minimize it in an attempt to protect them emotionally.

*On the very same day that I had my mammogram*, *my mother says that she wants to come a see the kids*. *We’re in the park*, *when she arrives*, *I have to tell her that everything’s fine and when we get home*, *I tell her everything*. *My mother’s devastated again and I tell her not to worry*, *that everything is going to be fine*. *(Patient 16)*

*Thinking about whether something could have been done differently*. Several women comment that, after their relapse, they think about whether the treatment was enough or there must have been something they could have done to avoid the relapse.

*You get furious*, *because you say*, *“I wasn’t supposed to get sick*, *because if*, *2 years ago when the first microcalcifications appeared I had had them removed*, *then I wouldn’t have metastasis*, *or maybe I would*. *(Patient 19)*

#### Metastatic breast cancer/time-limited chronic

*Re-interpreting the concept of metastasis*. Most of the participants in this stage state that they have had to give new meaning to the word, “metastasis,” since their first perception was directly related to death. In this way, they come to understand that cancer can become chronic, although they now have to take medication and go to the hospital on a regular basis. Nevertheless, they know that their life expectancy may be a few years. The women who are involved in a group point out how hard it is to see their fellow member pass away.

*What I now call my* “*new normal” consists of lots of visits to the hospital and never going back to work*. *(Patient18)*

They also state that at this stage, they do not identify with the disease generally known socially as “breast cancer”, where there is great emphasis placed on early detection and on their chances of being cured. This causes them to feel more isolated.

*These pink ribbon campaigns hurt us because they tend to underscore that everything is going to turn out fine because breast cancer has a very high cure rate; there is huge lack of awareness*. *(Patient 20)*

*Physical and emotional discomfort*. Most of the women in this stage report side effects from the treatments, although some comment that good quality-of-life can be preserved. On an emotional level, they say that they sometimes feel a certain agony due to not knowing how much longer the treatment will be effective. They live in a state of uncertainty that they try to cope with by focusing on their day-to-day and experience the good times deeply.

*When I’m not in pain*, *I try not to even remember what I have and go out and have fun with my family and live*. *(Patient 20)*

Several women who have children express with regret that they worry about their children enjoying them and remembering them when they were well. They are sad that they won’t be able to grow up in a normal family. Some also comment the impact this diagnosis is having on their partner.

*What I don’t want is for them to carry this baggage of having a sick mother*. *(Patient 18)*

A conflict with disability also appears, as many women report their desire to continue working, but feel that they can’t keep up with the pace of work. Additionally, several state that going through the medical board is a strenuous process, given that they look good physically.

*It’s hard to deal with*, *I’m a non-practicing lawyer and I have degrees galore*, *but I worked the first year and I couldn’t continue*. *(Patient 21)*

*Every year they call me again for the disability monitoring and they always threaten me*. *To be honest*, *the treatment doesn’t make me sick*, *but I don’t know how long it’s going to be like this*. *(Patient 19)*

*Social invisibility*. The participants say that they do not have any physical signs of being ill, that they look fine, although they know and feel that inside, they are not well. They say that it is sometimes hard to manage socially, since on occasion, they feel misunderstood and disparaged.

*I’m much sicker now*, *but people think or want to think that I’m fine*. *When I was doing chemo*, *it was like wearing a sign that said “cancer*.*” (Patient 17)*

## Discussion

This study describes the patient journey of women with breast cancer, specifying the different phases with the most relevant aspects of each, as well as the different cross-sectional features they report throughout the entire treatment process.

The results portray breast cancer as a process in which there is a striking feeling of loss of health and self-identity, changes in routines, personal and employment transformation, as well as emotional hardship during and after breast cancer treatment, aspects that are also reported in the literature [41, 42]. Earlier studies state that experiencing cancer is highly stressful. It involves a major threat to life or physical integrity, in addition to mental health, interfering with the path, projects, and plans patients have for their life over the short, medium, and, on occasion, long term as well [6]. Along with reporting adverse physical and psychological impacts, patients also report positive ways in which they have grown psychologically or emotionally from the experience [7, 42]. The diagnosis of breast cancer not only impacts the women individually, but also affects their surroundings. As reported in the literature, despite going through a very challenging time, the women struggle to put on a positive face and attempt to conserve the family’s well-being, specifically that of their children [7]. At the same time, the family is a fundamental source of support and usually provide indispensable support; however, it is not always effective, because family members do not fully understand the stresses involved in living with cancer [43]. Previous studies also reveal that for some women, their partners are one of their most significant supports; nonetheless, research also suggests that a cancer diagnosis predicts marital breakup more strongly for female survivors than males [44]. Our results reflect that the women frequently resort to other women in the same situation, possibly because they face significant unmet supportive care needs [30]. The need for social support may lead patients to seek social support groups consisting of people who are experiencing similar health crises, because such groups allow them to interact with those who best understand their suffering [43]. Another aspect that appears across the board is the relationship the participants have with the medical team. In this study, we have noted their trust in the medical team and acceptance of the treatments proposed without going into the clinical data of the disease and without needing to know the benefit provided by the treatment. Cancer patients are confronted with a potentially life-threatening [condition], feeling vulnerable, and need to rely heavily on their care providers, expecting the physician to act in their best interests [5]. Therefore, they need to have a close relationship, as well as comprehensive care [30]. Patients’ trust in a physician has been associated with a reduction of their fears and anxiety and [increased] satisfaction and adherence to treatment [5, 30]. We believe that it would be important to provide patients with accurate information, so as to avoid misunderstandings (such as cancer being synonymous with death, regardless of stage) as several participants in this study have reported, which can lead them to believe that the risk of relapse with and without chemotherapy is much greater than the oncologists estimate [45]. We believe that in future studies that it would be worthwhile to examine the peculiarities of each kind of patient information with the aim of determining how to break it up and make it both comprehensible and tolerable to promote patients’ well-being.

A breast cancer diagnosis is generally unexpected and practically all patients suffer psychological distress, such as feelings of uncertainty, disbelief, hopelessness, vulnerability, anger, fear, anxiety, and sadness [46, 47]. The literature has reported that many women experience peritraumatic distress or dissociation during the medical conversation in which they are given their diagnosis of cancer [48], which might account for the reactions of the respondents. Given that, when they receive their diagnosis, additional information is generally given to them, such as clinical aspects and preferred treatments. Repeating this information at subsequent appointments could contribute to improving communication with patient, since several participants stated that they found it hard to pay attention to the physician, given the emotional impact. Additionally, breast cancer patients tend to be diagnosed when they are relatively young, and often when they are in the middle furthering their career or raising children [12]. In spite of everything, the women try to put on as brave a face as they can and focus on maintaining their children’s well-being [7]. Telling children about their diagnosis is reportedly one of the biggest challenges; parents are usually unsure of how to tell them, because at the same time that they want it to be open and honest and cover their children’s developmental needs, they also want to protect them children [49].

Once diagnosed, breast cancer patients go through different treatments. The most salient experiences of these phases pertain to the impact of side effects on physical quality-of-life and psychological well-being, which is consistent with the literature [11]. Moreover, cancer therapy entails physical changes that affect their feminine identity, fertility, self-esteem, sexual functioning, and makes them more vulnerable [10, 50]. Women described their inner self as being on an emotional rollercoaster with highs and lows throughout the various phases of treatment [7]. Given treatment side effects and sequelae, these women are more likely to experience physical symptoms and psychological disorders than patients with other kinds of tumors [51]. The side effects involve an acute sense of loss of health and quality-of-life, as well as identity and femininity. It would be interesting for future research to explore the therapies used in grief counseling with cancer patients, as understanding and exploring this perspective could comprise an additional clinical aid.

Once the women have completed their treatments, they gradually get back to normal and many contemplate returning to work. However, in line with our results, the literature reveals that even though they want to normalize their lives, female breast cancer survivors feel that they will never return to their baseline status [7]. A significant number of patients experience difficulties in physical, cognitive, and emotional functioning after their treatment, such as symptoms like lymphedema, fatigue, pain, sleep disorders, cancer-related cognitive impairment, emotional stress, symptoms of depression and anxiety, problems with relationships, reduced sexual identity, fertility problems, and fear of cancer relapse [13, 14]. Furthermore, patients with hormone therapy suffer hot flashes, sweats, joint pain, weight gain, decreased libido, and low energy [52]. A sizeable number of these women also experience changes in employment status which can happen even 5–10 years following diagnosis [15]. Given that all these changes alter the structure of the woman’s everyday life, personalized care and treatment plans in cancer survivors are highlighted in the literature with extended specialized support being proposed that enables them to make a better psychosocially adjusted transition from treatment to follow-up [53] and advocating for the patient’s participation in all decisions that affect her during this period [54]. Further research is needed concerning how to structure the follow-up and support offered to these women during this stage so as to meet their needs and help them adjust to their new reality with the chronic sequelae caused by cancer and its treatment. On the other hand, the personal transformation of the initial stages of the journey are best seen during this phase. The literature shows that women who have had breast cancer report changes in their philosophy of life, such as embarking on a new life path, changing their priorities in life, as well as valuing life in general [42]. Most of the participants in our study place special emphasis on appreciating life, enjoying it more, and living each day to the fullest. Cancer survivors report being aware of how precarious life is, while also feeling the joy of being alive [55]. Similarly, they have been found to be more resilient and better able to repair their mood than healthy women [56].

About 5% of all patients with breast cancer are diagnosed when the disease is metastatic, whereas some 30% have suffered a relapse of an early breast cancer [17]. We saw that some women suffering a relapse after initial treatment with curative intent tend to wonder if the treatment was sufficient or if they should have done something more to prevent the relapse. Metastatic breast cancer is uncurable, which is why these women’s main psychosocial challenges are not the same as those who are diagnosed in early stages [18]. Faced with incurability, the women react with shock and fear of imminent death, but this anxiety diminishes once they begin treatment and learn that there are more treatment options [17]. During this phase, the interviewees reported impaired physical QoL and functioning, being hindered by pain, fatigue, or menopausal symptoms. Emotionally, they report suffering bouts of depression and anxiety, as well as fear because of the spread of their cancer. As for their relational QoL, their children’s welfare is their number one concern, especially for mothers of young children [17, 57]. What’s more, these women felt isolated from society in general and, more specifically, from the non-advanced breast cancer community, inasmuch as they feel that nobody understands what they are going through [18]. A psychosocial approach is especially important in this phase to help these women to continuously adapt to the changes of their individual clinical situation and to the progression of the disease, thereby improving their coping.

### Clinical implications

Having first-person information enables us to comprehend in detail the experiences of breast cancer patients, their situation, and emotional state, which favors holistic cancer care for health professionals.

Healthcare professionals should prepare women for a changed life situation, as well as to face prolonged, multimodal treatment (surgery, chemotherapy, hormone therapy, radiotherapy), and to confront physical and psychological sequelae, as well as the fear surrounding an uncertain prognosis. It is important to help them manage their expectations and fears and, to identify and address the issues and concerns that arise at different time points during treatment. The information and support offered should be adjusted to each woman’s individual needs, her life situation, her coping style, and the time and stage of their cancer. This more empathic, understanding outlook can also contribute to improving the physician-patient rapport, promoting communication, understanding, and shared decision-making.

Finally, a comprehensive understanding of the women’s psychosocial support endorses their belonging to groups of women with breast cancer, in which there is a relationship among equals. Further research is needed to specify the type needed so as to decrease both the impact of the death of women in the group, as well as the vast amount of information that they may end up obtaining, without needing it or requesting it.

### Limitations

This study was performed with Spanish participants, which is why certain aspects cannot reflect the experiences of breast cancer patients from other countries, given the particularities of both the Spanish healthcare system and Spanish culture. Likewise, the data attained were specific to women with breast cancer, which can scarcely be extrapolated to individuals with other cancers. Moreover, the findings do not reflect men’s experiences with breast cancer and research with this group would enrich the field further. In addition, the age of our participants ranged from 34 to 61 years; hence the results should be interpreted for a middle-aged population and do not reflect the experiences of women diagnosed at very early or very old ages. Finally, we believe that there may be a bias regarding the women who agree to participate, as this group has probably accepted their condition more, as well as having worked on it more.

Despite these limitations, we hope that our findings can contribute to better understanding the experiences of women with breast cancer.
